# Supplementation with calcium salts of cottonseed oil improves performance of *Bos indicus* animals consuming finishing diets^1^

**DOI:** 10.1093/tas/txaa080

**Published:** 2020-06-09

**Authors:** Marcos A A Carvalho, Bruno I Cappellozza, Bruna Silva, Thais S Castro, Marcos Renato Burim, Rafael C Cervieri

**Affiliations:** 1 Escola de Veterinária e Zootecnia, Universidade Federal de Goiás, Goiânia, GO, Brazil; 2 Nutricorp, Araras, SP, Brazil; 3 Fazenda Flórida, Guaiçara, SP, Brazil; 4 Nutribeef Consultoria, Botucatu, SP, Brazil

**Keywords:** *Bos indicus*, calcium salts of fatty acids, cottonseed byproducts, efficiency, feedlot, performance

## Abstract

Lipid ingredients are often used into feedlot cattle diets, primarily to increase energy density and improve efficiency parameters of the herd. Therefore, this study was designed to evaluate the effects of including calcium salts of fatty acids (CSFA) and increasing levels of cottonseed byproducts into feedlot diets. On day 0 of the study, 96 *Bos indicus* bullocks were individually weighed twice and initial body weight (BW) was considered the average of both measurements (initial BW = 287 ± 22.4 kg). Bulls were ranked by initial BW, allocated into 1 of 12 feedlot pens (eight bulls per pen), and pens randomly assigned to one of three treatments: 1) inclusion of 15.0% [dry matter (DM) basis] of cottonseed byproducts into the finishing diet (CTS-15; *n* = 4), 2) inclusion of 22.0% (DM basis) of cottonseed byproducts into the finishing diet (CTS-22; *n* = 4), and 3) inclusion of 2.7% (DM basis) of CSFA of cottonseed oil into the finishing diet (CSFA; *n* = 4). The experimental period lasted 135 d and consisted of 5 d of preadaptation, 15 d of adaptation (ADP), 31 d of growing (GRO), and 84 d of finishing (FIN). Performance and carcass characteristics data were evaluated at the end of the experimental period. A treatment × period interaction was observed on total DM intake (DMI; *P* < 0.0001), given that no treatment differences were observed during ADP (*P* > 0.33), whereas CSFA-supplemented animals had a reduced DMI during GRO and FIN phases (*P* < 0.05). When individual mean nutrient intake was evaluated, CSFA supplementation caused a reduction in crude protein and physically effective neutral detergent fiber intake (*P* ≤ 0.05), and tended to reduce metabolizable energy, net energy for maintenance and gain intake (*P* = 0.06). Additionally, CSFA inclusion or CTS increase into the diet did not affect final BW, BW change, average daily gain (ADG), hot carcass weight, carcass ADG, and yield gain (*P* ≥ 0.11). On the other hand, CSFA reduced DMI as percentage of BW and improved feed efficiency (FE; *P* < 0.02) and also tended to improve biological conversion (BC; *P* = 0.07) versus CTS. Similarly, increasing CTS byproducts in the diet improved FE and BC (*P* = 0.02) but also tended to increase dressing percentage (DP; *P* = 0.08). In summary, including CSFA into feedlot diets reduced DMI but improved FE and BC of beef cattle, demonstrating the efficacy of this technology on feedlot beef cattle diets. Moreover, increasing cottonseed byproducts into the diets also benefited FE, BC, and DP of finishinw *B. indicus* cattle.

## INTRODUCTION

Lipid feedstuffs are often included into feedlot diets to increase their energy density and to improve feed efficiency (FE) of the herd [National Academies of Sciences, Engineering, and Medicine (NASEM), 2016]. More specifically, in Brazil, given the high availability and low cost, cottonseed byproducts are largely employed in beef cattle diets as lipid sources. In a recent survey with Brazilian nutritionists and feedlot consultants, Pinto and Millen (2016) demonstrated that 82.7% of the participants included cottonseed byproducts into beef cattle feedlot diets as lipid sources, whereas whole cottonseed (WCS) is the primary feedstuff used (72.4% of participants). Whole cottonseed is a byproduct that supplies fiber, lipid, and protein (Cranston et al., 2006), but one potential problem related to WCS feeding is gossypol toxicity and its effects on quality characteristics of edible products, such as meat and milk.

Another feasible lipid alternative to be used into feedlot diets is calcium salts of fatty acids (CSFA), given that its partial protection against rumen biohydrogenation alleviates the negative effects that lipids might induce into the rumen, such as toxicity to gram-positive bacteria and protozoa, biofilm effect into the feed particles, and a reduction in rumen organic matter (OM) and dry matter (DM) degradability (Hess et al., 2008), as well as in DM intake (DMI; NASEM, 2016). In fact, Pinto and Millen (2016) demonstrated that CSFA are the second lipid source used in feedlot diets. To the best of our knowledge, no other study evaluated the effects of CSFA of cottonseed oil in feedlot diets containing increasing levels of cottonseed byproducts. Hence, we hypothesized that including CSFA of cottonseed oil would improve efficiency of the herd versus diets containing increased levels of cottonseed byproducts. Therefore, our objective was to evaluate the effects of 1) increasing levels of cottonseed byproducts and 2) CSFA inclusion on performance of *B. indicus* beef animals receiving a finishing high-concentrate diet.

## MATERIALS AND METHODS

The present experiment was conducted at a commercial feedlot operation (Fazenda Flórida), located in Guaiçara, São Paulo, Brazil (21°37’33” S, 49°47’52” W, and elevation of 437 m) from September 2018 to January 2019. All animals utilized herein were cared for in accordance with the practices outlined in the Guide for the Care and Use of Agricultural Animals in Agricultural Research and Training (FASS, 2010).

### Animals and Diets

On day −1 of the study, 96 *Bos indicus* Nellore beef bulls were road-transported from the commercial farm to the feedlot facility (approximately 170 km) and allowed to rest for 16 h at the working facility with ad libitum access to *Brachiaria decumbens* hay and water. On day 0, all animals were individually weighed twice, approximately 60 min apart, and initial body weight (BW) was considered the average of both measurements (initial BW = 287 ± 22.4 kg; initial age 23 ± 3.6 mo). Additionally, bulls were vaccinated against respiratory (5 mL/head; Biopoligen HS; Biogenesis Bagó SA, Buenos Aires, Argentina) and clostridium (5 mL/head; Fortress 7; Zoetis, São Paulo, SP, Brazil) pathogens and administered an anthelmintic (1 mL/50 kg BW; Dectomax, Zoetis). Bulls were ranked by initial BW, allocated into 1 of 12 feedlot pens (eight bulls per pen), and pens randomly assigned to one of three treatments: 1) inclusion of 15% (DM basis) of cottonseed byproducts into the finishing diet (CTS-15; *n* = 4), 2) inclusion of 22% (DM basis) of cottonseed byproducts into the finishing diet (CTS-22; *n* = 4), and 3) inclusion of 2.7% (DM basis) of CSFA (Nutri Gordura; Nutricorp, Araras, SP, Brazil) into the finishing diet (CSFA; *n* = 4). The CSFA used herein consisted of cottonseed oil and contained 95.0% DM, 0.8% crude protein (CP), 82.5% ether extract (EE), 1.0% neutral detergent fiber (NDF), 6.7% calcium (Ca), 190% total digestible nutrients (TDN), 6.95 and 5.30 Mcal/kg of net energy for maintenance and gain (NEm and NEg), respectively. Diets were formulated to be isocaloric, isonitrogenous, isofiber (NDF and physically effective NDF; peNDF), offered twice a day (0800 and 1500 hours) in a manner to ensure ad libitum intake and to result in 5% orts. Moreover, throughout the experimental period, all animals had full access to water and were maintained in open-sided pens with dirt-based floors (18 × 5 m and 1.0 m of linear feedbunk per animal).

The experimental period lasted 135 d and consisted of 5 d of preadaptation, 15 d of adaptation (ADP), 31 d of growing (GRO), and 84 d of finishing (FIN). The preadaptation was required to avoid metabolic disorders, acclimate animals to the facility, personnel, and diet, and to minimize stress at feedlot entry. Hence, from day 0 to 4, they were offered 1.0 and 5.0 kg/d of cottonseed meal and sugarcane silage, respectively (OM basis). Furthermore, Table 1 describes the composition and nutritional profile of the diets used during ADP, GRO, and FIN.

**Table 1.** Composition and nutritional profile of the diets offered to the animals during the experiment* ^,†^

	ADP			GRO			FIN		
Item	CTS-15	CTS-22	CSFA	CTS-15	CTS-22	CSFA	CTS-15	CTS-22	CSFA
Composition, % DM									
Sugarcane silage	25.30	25.30	27.17	19.57	18.43	22.55	16.37	13.13	19.10
Ground corn	51.28	51.28	54.35	60.67	59.87	60.94	64.74	61.53	63.90
Whole cottonseed	13.68	13.68	5.80	11.74	11.51	5.18	11.16	15.00	6.80
Cottonseed meal	4.79	4.79	5.80	3.91	6.45	4.88	3.72	6.97	3.10
Urea	1.37	1.37	1.67	1.11	0.74	1.39	1.01	0.37	1.40
Nutri Gordura	–	–	1.67	–	–	2.07	–	–	2.70
Mineral–vitamin mix	3.58	3.58	3.54	3.00	3.00	2.99	3.00	3.00	3.00
Nutritional profile, % DM									
DM	64.8	64.8	62.7	69.0	70.0	65.6	71.8	75.0	68.2
CP	15.0	15.0	14.9	14.0	13.9	13.9	13.6	13.7	13.6
RDP	9.5	9.5	9.6	8.4	7.9	8.5	8.0	7.5	8.3
EE	5.3	5.3	5.3	5.1	5.1	5.6	5.1	5.8	6.5
NDF	30.5	30.5	28.7	26.3	26.2	25.6	24.1	24.5	23.7
peNDF	22.7	22.7	20.2	18.8	18.3	17.4	16.8	17.1	16.1
Starch	38.1	38.1	40.2	44.7	44.1	44.8	47.5	45.2	46.8
TDN^‡^	74.1	74.1	74.4	77.0	77.2	77.4	78.2	79.5	79.7
ME, Mcal/kg**	2.74	2.74	2.75	2.85	2.86	2.86	2.89	2.94	2.95
NEm, Mcal/kg**	1.82	1.82	1.83	1.91	1.92	1.92	1.95	1.99	1.99
NEg, Mcal/kg**	1.20	1.20	1.21	1.27	1.28	1.28	1.31	1.34	1.34

ME, metabolizable energy; RDP, rumen degradable protein.

*Experimental period lasted 135 d.

^†^ADP = 15 d; GRO = 31 d; FIN = 84 d.

^‡^Calculated based on equations described by Weiss et al. (1992).

**Calculated based on equations described by NASEM (2016).

### Sampling

Individual animal BW was collected twice at the beginning (day 0) and end of the experimental period (day 135) in order to calculate BW change and average daily gain (ADG) during the experiment. Total DMI and individual nutrient intake were evaluated on a daily basis throughout the experimental period by weighing the feed offered and refused in the following day. At the end of the experimental period, total BW gain and total DMI were used for the calculation of the FE, whereas mean BW was used for determination of DMI as a percentage of BW.

Samples of ingredients were collected at the beginning of the experiment and analyzed for nutrient concentration by a commercial laboratory (3RLab; Lavras, MG, Brazil). All samples were analyzed in duplicates by wet chemistry procedures for concentrations of CP [method 984.13; Association of Official Analytical Chemists (AOAC), 2006], NDF (Van Soest et al., 1991; modified for use in an Ankom-200 fiber analyzer; Ankom Technology Corp., Fairport, NY), and acid detergent fiber (ADF; method 973.18 modified for use in an Ankom-200 fiber analyzer; Ankom Technology Corp.; AOAC, 2006). Samples were also analyzed for EE content according to procedures described by AOAC (2006). Moreover, calculation of TDN concentration was performed according to equations proposed by Weiss et al. (1992), whereas NEm and NEg were calculated according to equations described by NASEM (2016). After the collection, samples were immediately frozen at −20 °C until further shipment and laboratorial analysis.

At the end of the experimental period, animals were road-transported and slaughtered in a commercial packing plant located approximately 5 km away from the feedlot facility (JBS; Lins, SP, Brazil). Hot carcasses were separated into two symmetrical sections, weighed to obtain hot carcass weight (HCW), and individually identified. Dressing percentage (DP) was calculated by dividing HCW by final BW of the animal on day 135 of the study. Initial DP was estimated in 50% and then it was calculated as the amount of carcass gained by the animals during the experiment. Carcass ADG was calculated by dividing the carcass gain and the number of days on feed (135 d), whereas yield gain (YG) was calculated by dividing carcass ADG and ADG. Additionally, biological conversion (BC) was determined by dividing total DMI of each pen by 15-kg carcass produced during the experiment.

### Statistical Analysis

For all analysis, pen was considered the experimental unit. All data were analyzed using the PROC MIXED procedure of SAS (version 9.4; SAS Inst. Inc.; Cary, NC) and Sattherwaite approximation to determine the denominator df for the test of fixed effects. For total DMI, the model statement contained the fixed effects of treatment, period (ADP, GRO, and FIN), and the resultant interaction. Data were analyzed using pen (treatment) as random variable. For all other performance data, model statement contained the fixed effects of treatment and data were analyzed using animal (pen) and pen (treatment) as random variables. Orthogonal contrasts were used for partition-specific treatments as follows: 1) no CSFA versus CSFA and 2) CTS-15 versus CTS-22. All results are reported as least-square means and separated using PDIFF. Significance was set at *P* ≤ 0.05 and tendencies were denoted if 0.05 < *P* ≤ 0.10. Results are reported according to main effects if no interactions were significant or according to the highest-order interaction.

## RESULTS

**Figure 1.**
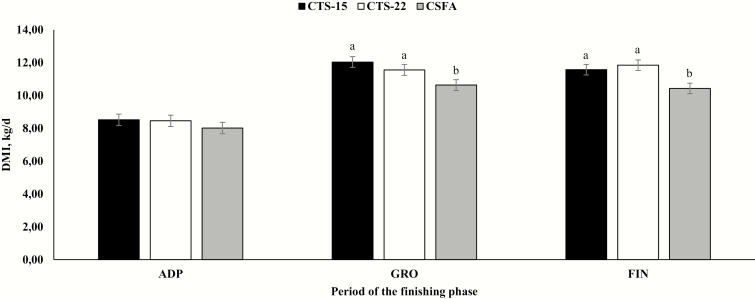
Mean DMI of *Bos indicus* beef animals receiving diets containing either 15% (CTS-15; *n* = 4) or 22% (CTS-22; *n* = 4) of cottonseed byproducts or calcium salts of fatty acids of cottonseed oil (CSFA; *n* = 4). A treatment × period was observed (*P* < 0.0001). Within periods, different letters denote differences (*P* ≤ 0.05).

A treatment × period interaction was observed for total DMI (*P* < 0.0001; Fig. 1). No treatment differences were observed during ADP (*P* > 0.33), whereas CSFA-supplemented animals had a reduced DMI during GRO and FIN phases (*P* < 0.05; Fig. 1). No further differences among CTS-15 and CTS-22 were observed for GRO and FIN (*P* ≥ 0.68; Fig. 1). When individual mean nutrient intake was evaluated, CSFA supplementation caused a reduction in CP and peNDF intake (*P* ≤ 0.05) and tended to reduce ME, NEm, and NEg intake (*P* = 0.06; Table 2). On the other hand, increasing CTS byproducts inclusion into feedlot diets by 50% did not alter nutrient intake (*P* ≥ 0.38; Table 2).

**Table 2.** DMI and nutrient intake of *Bos indicus* beef animals receiving diets containing either 15% (CTS-15; *n* = 4) or 22% (CTS-22; *n* = 4) of cottonseed byproducts or calcium salts of fatty acids of cottonseed oil (CSFA; *n* = 4)*

	Treatments				Contrasts	
Item	CTS-15	CTS-22	CSFA	SEM	No CSFA vs. CSFA	CTS-15 vs. CTS-22
Nutrient intake, kg/d						
DM	10.71	10.62	9.69	0.320	0.05	0.85
CP	1.51	1.50	1.36	0.044	0.04	0.85
EE	0.55	0.57	0.56	0.017	0.94	0.38
peNDF	2.02	2.02	1.72	0.056	<0.01	0.97
TDN	8.21	8.20	7.50	0.250	0.06	0.97
Energy intake, Mcal/d						
ME	30.4	30.3	27.7	0.92	0.06	0.99
NEm	20.4	20.4	18.6	0.62	0.06	0.98
NEg	13.6	13.6	12.4	0.42	0.06	0.90

ME, metabolizable energy.

*Experimental period lasted 135 d.

No treatment effects were observed for initial BW (*P* > 0.81), indicating that similar management animals were reared prior to the beginning of the study. Additionally, CSFA inclusion or CTS increase did not affect final BW, BW change, ADG, HCW, carcass ADG, and YG (*P* ≥ 0.11; Table 3). Conversely, CSFA inclusion reduced DMI as percentage of BW and improved FE (*P* < 0.02) and also tended to improve BC (*P* = 0.07; Table 3). Similarly, increasing CTS byproducts in the diet improved FE and BC (*P* = 0.02) but also tended to increase DP (*P* = 0.08; Table 3).

**Table 3.** Performance and carcass characteristics of *Bos indicus* beef animals receiving diets containing either 15% (CTS-15; *n* = 4) or 22% (CTS-22; *n* = 4) of cottonseed byproducts or calcium salts of fatty acids of cottonseed oil (CSFA; *n* = 4)*

	Treatments				Contrasts	
Item	CTS-15	CTS-22	CSFA	SEM	No CSFA vs. CSFA	CTS-15 vs. CTS-22
Performance						
Initial BW, kg	287.2	287.2	287.7	1.67	0.81	0.99
Final BW, kg	514.5	526.4	508.1	7.50	0.23	0.30
BW change, kg	227.3	239.2	220.4	7.36	0.21	0.29
DMI, % BW	2.87	2.80	2.57	0.057	<0.01	0.37
ADG, kg/d	1.684	1.772	1.632	0.0545	0.21	0.29
FE, g/kg	153	163	167	2.4	0.02	0.02
Carcass						
HCW, kg	270.8	281.4	271.9	4.81	0.51	0.17
DP, %	52.6	53.4	53.4	0.33	0.32	0.08
Carcass ADG, kg/d	0.942	1.021	0.949	0.0350	0.48	0.16
YG, %	56.0	57.6	58.1	0.08	0.20	0.11
BC, DMI/15-kg carcass	177.0	160.0	158.5	3.74	0.07	0.02

*Experimental period lasted 135 d.

## DISCUSSION

The primary goals of the experiment were to evaluate the effects of 1) including a CSFA of cottonseed oil and 2) increasing levels of cottonseed byproducts into feedlot diets of *B. indicus* animals. This objective arose from the fact that cottonseed byproducts are widely available at an attractive cost in Brazil and that feedlot nutritionists often use cottonseed byproducts and CSFA as lipid sources into feedlot diets (Pinto and Millen, 2016). To the best of our knowledge, no other study in the literature compared CSFA of cottonseed oil with cottonseed byproducts as lipid sources for feedlot *B. indicus* cattle.

Although experimental diets were formulated to be isofiber based on NDF and peNDF contents, the quality of the fiber-rich source might also affect DMI of beef cattle. Therefore, the presence of linter, a highly fermentable OM in whole cottonseed, might have increased ruminal DM degradation rate compared with sugarcane silage, for example (Bo et al., 2012). In agreement with our results, others have reported that CSFA supplementation has been shown to reduce total DMI (kilograms or percentage of BW) and individual nutrient intake (Allen, 2000). Reviewing possible factors impacting hepatic oxidation in ruminants, Allen et al. (2009) demonstrated that unsaturated fatty acids (FA) are more potent in stimulating hepatic oxidation, which, in turn, will cause a greater DMI reduction versus saturated FA. Hence, it can be speculated that ruminal biohydrogenation is reduced when CSFA is supplemented and more unsaturated FA are able to reach the small intestine for absorption (Sukhija and Palmquist, 1990). Nonetheless, Hales et al. (2017) suggested that an adaptation on digestible energy:metabolizable energy (DE:ME) ratio should be taken into account when lipid-enriched diets (>6.0% EE) are offered to cattle. In other words, these authors suggested that the coefficient value of 0.92 could be used instead of the well-known and broadly adopted 0.82. If we take this adjustment in consideration, CSFA-supplemented animals had intakes of ME, NEm, and NEg of 30.5, 20.4, and 12.4 Mcal/d, respectively.

Additionally, CSFA supplementation or linear increases on CTS byproducts did not impact final BW and ADG, supporting the fact that diets were formulated to be isocaloric and isonitrogenous. Nonetheless, supplementation with CSFA improved efficiency variables of the beef cattle herd, such as FE and BC. These results might be explained by the greater DE:ME ratio aforementioned, greater amount of ME available to the animals, and the greater efficiency by which CSFA sources are transferred and deposited into the animal when compared with other nonprotected lipids, as well as protein- and energy-based feedstuffs (Zinn and Shen, 1996; Hales et al., 2017). These results are even more important in a scenario with drastic variation on ingredient costs (ton or point of nutrient), which could lead to changes on performance and efficiency of the herd and, consequently, impact profitability of the feedlot operation. Lastly, the effects of CSFA on DP and carcass characteristics of beef cattle are still scarce, variable, and warrant further investigation by researchers.

Recently, Gouvêa et al. (2020) evaluated the effects of linearly increasing whole cottonseed as replacement for ground corn into the diets of finishing bulls. These authors reported that DMI reduced in the beginning of the finishing period as whole cottonseed on the diet increased, whereas no negative effects were detected on ADG and FE. Moreover, greater carcass characteristics were observed when whole cottonseed inclusion ranged from 2.2% to 10.3% diet DM. Conversely, 15% whole cottonseed inclusion increased FE of finishing bulls when the total 133-d feeding period was analyzed. In the present study, increasing cottonseed byproducts as a manner to increase EE content of the diet yielded positive results on efficiency parameters (BC and FE) and DP of finishing *B. indicus* bullocks but did not impact total DMI and nutrient intake at ADP and GRO phases.

In summary, including calcium salts of fatty acids since the beginning of the feedlot phase (1.67%, 2.07%, and 2.70% in ADP, GRO, and FIN phases) caused a reduction in DMI and nutrient intake but improved FE and BC of beef cattle receiving high-concentrate diets containing cottonseed byproducts, demonstrating the efficacy and benefits of this technology on feedlot diets offered to Nellore cattle. Moreover, increasing cottonseed byproducts into the diets also benefited FE, BC, and DP of finishing *B. indicus* cattle.
